# Metal-Induced Fluorescence Quenching of Photoconvertible Fluorescent Protein DendFP

**DOI:** 10.3390/molecules27092922

**Published:** 2022-05-03

**Authors:** In Jung Kim, Yongbin Xu, Ki Hyun Nam

**Affiliations:** 1 Division of Biotechnology, College of Life Sciences and Biotechnology, Korea University, Seoul 02841, Korea; ij0308@korea.ac.kr; 2 Research Institute of Tailored Food Technology, Kyungpook National University, Daegu 41566, Korea; 3 Department of Bioengineering, College of Life Science, Dalian Minzu University, Dalian 116600, China; yongbinxu@dlnu.edu.cn; 4 Key Laboratory of Biotechnology and Bioresources Utilization of Ministry of Education, Dalian Minzu University, Dalian 116024, China; 5 Department of Life Science, Pohang University of Science and Technology, Pohang 37673, Korea; 6 POSTECH Biotech Center, Pohang University of Science and Technology, Pohang 37673, Korea

**Keywords:** metal biosensor, fluorescent protein, DendFP, fluorescence quenching, crystal structure

## Abstract

Sensitive and accurate detection of specific metal ions is important for sensor development and can advance analytical science and support environmental and human medical examinations. Fluorescent proteins (FPs) can be quenched by specific metal ions and spectroscopically show a unique fluorescence-quenching sensitivity, suggesting their potential application as FP-based metal biosensors. Since the characteristics of the fluorescence quenching are difficult to predict, spectroscopic analysis of new FPs is important for the development of FP-based biosensors. Here we reported the spectroscopic and structural analysis of metal-induced fluorescence quenching of the photoconvertible fluorescent protein DendFP. The spectroscopic analysis showed that Fe^2+^, Fe^3+^, and Cu^2+^ significantly reduced the fluorescence emission of DendFP. The metal titration experiments showed that the dissociation constants (*K*_d_) of Fe^2+^, Fe^3+^, and Cu^2+^ for DendFP were 24.59, 41.66, and 137.18 μM, respectively. The tetrameric interface of DendFP, which the metal ions cannot bind to, was analyzed. Structural comparison of the metal-binding sites of DendFP with those of iq-mEmerald and Dronpa suggested that quenchable DendFP has a unique metal-binding site on the β-barrel that does not utilize the histidine pair for metal binding.

## 1. Introduction

Heavy metals contamination is a serious problem for human health as they do not biodegrade and are eliminated at a slow rate by ecological systems. Depending on their dose and chemical form, heavy metals can have varied effects [1,2]. The detection and identification of heavy metals is important to the field of biomedical science and for environmental monitoring [3]. Heavy metal ions can be detected using fluorescence spectroscopy, UV-vis absorption, atomic absorption, inductively coupled plasma (ICP) emission spectroscopy, and voltammetry [3]. Moreover, metal detection using biomaterials, such as peptides, proteins, enzymes, antibodies, nucleic acids, and whole cells, has also been reported [4]. Among them, fluorescence emission spectroscopy is an attractive approach due to its high sensitivity [3]. This allows for the detection of very small amounts of heavy metals using fluorescence probes.

Fluorescent proteins (FPs) are highly sensitive optical markers with spatial and temporal specificity that are widely used to analyze the function of target molecules in molecular or cellular biology [5,6,7,8,9,10]. GFP (green fluorescent protein)-like FPs commonly have a β-barrel structure composed of 11 β-strands, within which tripeptides form chromophores through folding, cyclization, dehydration, and oxidation [5]. Each FP not only has unique spectroscopic characteristics but also has the ability to change its fluorescence characteristics according to the external environment, including conditions such as pH and temperature [11,12,13]. In particular, FPs display a fluorescence-quenching phenomenon by a specific metal, and these spectroscopic features can be used to develop FP as a receptor, which can then be used as a metal biosensor [14,15,16,17,18,19,20]. FPs are expressed intracellularly, are soluble in the cytoplasm, and do not require an additional cofactor to fluoresce [5,7]. In addition, it has the advantage of high sensitivity, even at low concentrations of FP, and is easily detected with a fluorescence microscope or spectroscopy [5,7].

**Figure 1:**
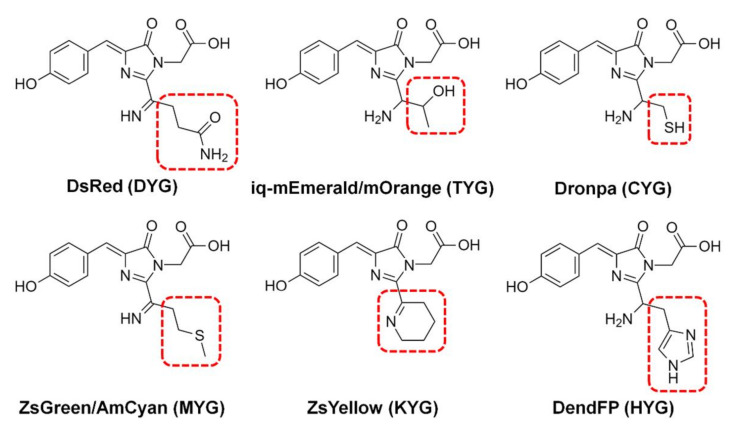
Chromophores of fluorescent proteins showing fluorescence quenching by metals. The chromophore tripeptide sequence is shown in parentheses.

The fluorescence quenching of various FPs, such as BFPms1, DsRed, iq-mEmerald, Dronpa, AmCyan, mOrange, ZsYellow, and ZsGreen, by metal ions has been analyzed via spectroscopic or structural studies (Figure 1) [16,17,18,19,21,22,23,24,25]. Although several FPs demonstrate similar properties regarding fluorescence quenching by metal ions overall, they all have different sensitivities for fluorescence quenching depending on the specific metal type. For example, spectroscopic analysis shows that Dronpa, AmCyan, mOrange, and ZsYellow are highly sensitive to Cu^2+^ [17,18,19], whereas ZsGreen is more sensitive to Fe^2+^ and Fe^3+^ than to Cu^2+^ [25]. Meanwhile, the fluorescence of these quenched FPs can be recovered by adding metal chelators, such as ethylenediaminetetraacetic acid (EDTA), when the metal ions are located on the external area of the β-barrel [17].

FPs display a variety of quenching mechanisms. In BFPms1, the metal is bound directly to the chromophore [24], whereas on the β-barrel of iq-mEmerald and Dronpa, the metal ions are bound to the surface [17,23]. In contrast, fluorescence quenching of ZsYellow is suggested to occur due to the close distance between the chromophore and metal ions at high metal ion concentrations, without the binding of specific metals to the protein [19]. Therefore, metal-induced fluorescence quenching of FPs shows diverse spectral properties and quenching mechanisms. To develop FP-based metal biosensors, it is necessary to extensively analyze the various spectroscopic properties of a wide range of new FPs to create various libraries.

The DendFP from *Dendronephthya* sp is a member of the Kaede-like group of photoconvertible fluorescent proteins [26,27]. This FP is irreversibly converted from a green to red fluorescent state following irradiation with light from the UV region of the spectrum. The excitation/emission maxima of the green and red state of DendFP are 492/508 and 555/575 nm, respectively [27]. The fluorescence quantum yields of the green and red states of DendFP are 0.76 and 0.64, respectively [27]. This protein and its monomeric variant are widely used as optical markers in the cellular and molecular biology fields [28,29,30], but its metal-induced fluorescence quenching has not yet been characterized. Herein, we report the spectroscopic and structural analysis of the metal-induced fluorescence quenching of the fluorescent photoconversion protein DendFP. Spectroscopic study shows that the fluorescence emission of DendFP was quenched by the addition of Fe^2+^, Fe^3+^, and Cu^2+^. Metal-titration and reversibility of quenched DendFP were performed to further confirm the effects of Fe^2+^, Fe^3+^, and Cu^2+^ on the fluorescence properties of DendFP. Structural comparison of the metal-binding sites of DendFP with those of other FPs suggested that DendFP has a novel metal-binding site. Our results shed light on the spectral properties of the metal-induced fluorescence quenching of DendFP.

## 2. Results

### 2.1. Fe^2+^-, Fe^3+^-, and Cu^2+^-Induced Fluorescence Quenching

**Figure 2:**
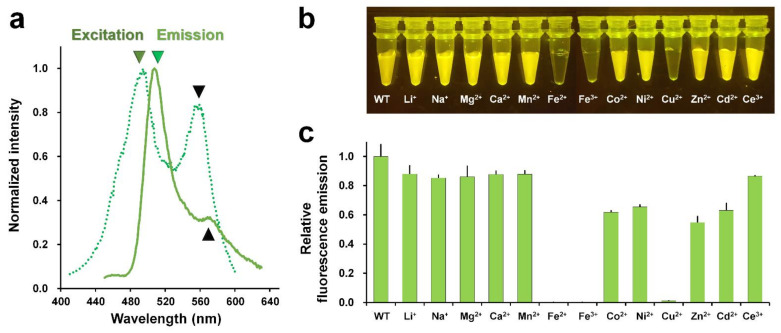
Metal screen for quenching DendFP. (**a**) Verification of purified DendFP by fluorescence spectral scanning. The maximum peaks of fluorescence excitation (forest, dot-line) and emission (green, line) are 494 and 507 nm, respectively. (**b**) Visualization of the metal-induced fluorescence quenching of DendFP. The sample mixtures containing DendFP (10 μM, 50 μL) and each of the metal ions (10 mM, 50 μL) were exposed to LED light (470 nm). (**c**) Relative fluorescence emission intensity of DendFP in the presence of various metal ions.

The maximum fluorescence excitation and emission wavelengths of the purified DendFP were scanned to validate the purified DendFP. The maximum peak wavelengths for the excitation and emission of DendFP were 494 and 507 nm, respectively (Figure 2a); these wavelengths of excitation and emission were blue-shifted by 2 and 1 nm, respectively, when compared with those of the original DendFP (λ_ex_ = 492 nm and λ_em_ = 508 nm) [27]. Meanwhile, we observed a shoulder peak at 559 nm in the fluorescence emission spectra; this was considered to be the partially photoconverted state of DendFP (Figure 2a).

Next, metal-induced fluorescence quenching of DendFP was investigated using Li^+^-, Na^+^-, Mg^2+^-, Ca^2+^-, Mn^2+^-, Fe^2+^-, Fe^3+^-, Co^2+^-, Ni^2+^-, Cu^2+^-, Zn^2+^-, Cd^2+^-, and Ce^3+^-containing solutions. The mixture of DendFP and metal ions was initially visualized following exposure to LED light at 470 nm (Figure 2b). As a result, the fluorescence intensity of DendFP decreased significantly in the mixtures containing Fe^2+^, Fe^3+^, and Cu^2+^. Next, the fluorescence intensities of these mixtures were quantitatively analyzed at an emission wavelength of 530 nm (Figure 2c). When compared with the emission value of apo DendFP, the addition of Fe^2+^, Fe^3+^, and Cu^2+^ significantly reduced the fluorescence emission by 99.94, 99.89, and 98.81%, respectively. Co^2+^, Ni^2+^, Zn^2+^, and Cd^2+^ also reduced the fluorescence intensity by 38.25%, 34.60%, 46.51%, and 36.88%, respectively. Li^+^, Na^+^, Mg^2+^, Ca^2+^, Mn^2+^, and Ce^3+^ induced a modest reduction in the fluorescence intensity of 12.11%, 14.82%, 13.99%, 12.31%, 12.23%, and 13.46%, respectively. Therefore, the fluorescence quenching of DendFP was highly sensitive to quenching by Fe^2+^, Fe^3+^, and Cu^2+^.

### 2.2. Titration of DendFP with Quenchable Metal Ions

**Figure 3:**
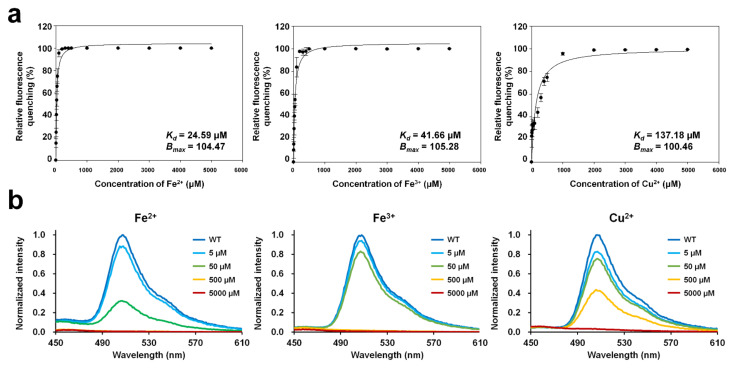
Metal titration of quenchable DendFP and fluorescence emission spectra in the presence of metal ions. (**a**) DendFP was titrated with various concentrations of Fe^2+^, Fe^3+^, and Cu^2+^ (0 to 1 mM, resulting in final concentrations of 0 to 5000 μM). The data fitting was performed with the one metal-binding mode by the Langmuir equation using SigmaPlot. Data represent means ± standard deviations of five replicates. (**b**) The fluorescence emission spectra of DendFP in the presence of Fe^2+^, Fe^3+^, or Cu^2+^ at different concentrations (0 to 5000 μM).

To determine the dissociation constant (*K*_d_) and maximum binding capacity (*B*_max_) of the metal ions with DendFP, 4 μM DendFP was incubated with various concentrations of Fe^2+^, Fe^3+^, and Cu^2+^ (Figure 3a). In the Fe^2+^ titration, 50, 500, and 5000 μM Fe^2+^ reduced the fluorescence of DendFP by 74.87%, 99.93%, and 99.94%, respectively (Figure 3a). At 5 μM Fe^2+^, the fluorescence intensity was reduced by 15.14%. In the Fe^3+^ titration, 50, 500, and 5000 μM Fe^3+^ reduced the fluorescence intensity of DendFP by 55.00%, 99.80%, and 99.93%, respectively (Figure 3a). When 5 μM Fe^3+^ was present, the fluorescence intensity was reduced by 10.53%. In the Cu^2+^ titration, 500 and 5000 μM Cu^2+^ reduced the fluorescence emission of DendFP by 74.68% and 99.92%, respectively (Figure 3a). When 5 and 50 μM Cu^2+^ were present, the fluorescence intensities were reduced by 22.81 and 33.07%, respectively. The fluorescence emission spectra of DendFP in the presence of Fe^2+^, Fe^3+^, or Cu^2+^ at different concentrations did not display any shift in wavelength (Figure 3b).

**Figure 4:**
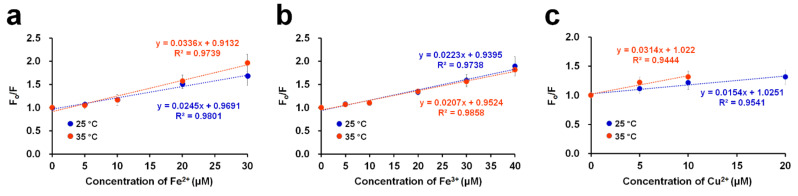
Stern–Volmer plots of the DendFP quenching system by (**a**) Fe^2+^, (**b**) Fe^3+^, and (**c**) Cu^2+^ at 2 different temperatures: 25 and 35 °C.

In the various applications of fluorescence quenching of a molecule, binding constants, such as *K*_d_ and *B*_max_, have been determined based on the Langmuir isotherm to elucidate the metal ion-binding characteristics of the hepatitis C virus RNA polymerase [31], the properties of ionic liquids binding to human serum albumin [32], and the fluorescent protein–metal ion interaction [18]. In the present study, the Langmuir isotherm was also used to determine the binding constants to investigate the interaction between DendFP and metal ions. The metal titration results showed that *K*_d_ of Fe^2+^, Fe^3+^, and Cu^2+^ for DendFP was 24.59, 41.66, and 137.18 μM, respectively. *B*_max_ of Fe^2+^, Fe^3+^, and Cu^2+^ for DendFP was 104.47, 105.28, and 100.46, respectively. Taken together, the dissociation constant of Fe^3+^ for DendFP was 1.69-fold higher than that of Fe^2+^, and the dissociation constant of Cu^2+^ for DendFP was 5.58- and 3.29-fold higher than those of Fe^2+^ and Fe^3+^, respectively. Based on the dissociation constant, DendFP quenching could be enhanced by metal ions in the following order: Fe^2+^ > Fe^3+^ >> Cu^2+^. Although the fluorescence-quenching sensitivity of Fe^2+^ towards DendFP was a little bit higher that of Fe^3+^, these values were comparable when compared to that of Cu^2+^ in the spectroscopic results. Consequently, it is considered that the oxidation state of iron did not pronouncedly affect fluorescence quenching. This is consistent with the result of Figure 4a,b, in which the *K*_SV_ values for Fe ions obtained at 25 °C are similar.

To examine the quenching mechanism, the Stern–Volmer equation was used [33]:

*F*_o_/*F* = 1 + *K*_SV_[Q]

where *F*_o_ and *F* represent the fluorescence intensities in the absence and presence of (different concentrations) metal ions ([Q]), respectively, and *K*_SV_ represents the Stern–Volmer constant. By constructing a linear plot of the relative quenching ratio (*F*_o_/*F*) as a function of the quencher concentration (i.e., metal ions), the *K*_SV_ value, as the slope, can be obtained. In this study, quenching experiments were conducted with DendFP in the presence of metal quenchers in the linear range at 25 and 35 °C (Figure 4). In the cases of Fe^2+^ and Cu^2+^, higher values of *K*_SV_ were obtained at 35 °C. To be specific, the *K*_SV_ values were 0.0245 × 10^6^ and 0.0336 × 10^6^ L mol^−1^ at 25 and 35 °C, respectively, for Fe^2+^ while the values were 0.0154 × 10^6^ and 0.0314 × 10^6^ L mol^−1^ at 25 and 35 °C, respectively, for Cu^2+^. These results imply that the fluorescence quenching of DendFP by these metals occurs via dynamic (or collisional) processes. However, in the case of Fe^3+^, increasing the temperature did not cause a noticeable difference in the value.

### 2.3. Reversibility of Metal-Induced Quenching

**Figure 5:**
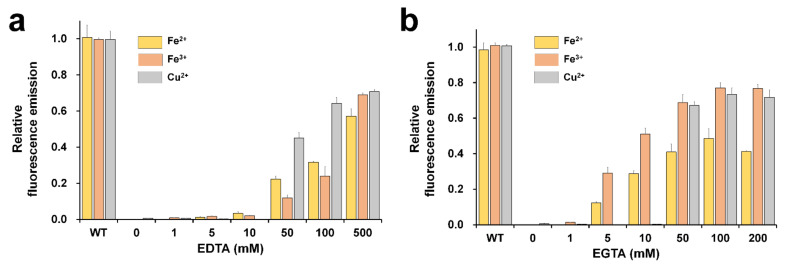
Reversibility of DendFP fluorescence quenching. Various concentrations of (**a**) EDTA or (**b**) EGTA were added (as chelators) to the DendFP samples quenched using Fe^2+^, Fe^3+^, and Cu^2+^. Data represent the means ± standard deviations of three replicates.

The reversibility test of fluorescence-quenched FPs is important in terms of whether FP is sustainable for application as a biosensor probe [19]. In addition, it provides information on whether the metal is inside or outside the β-barrel of the FP’s structure [18]. To investigate the reversibility of fluorescence, DendFP was initially quenched by 1 mM Fe^2+^, 1 mM Fe^3+^, or 10 mM Cu^2+^ solution. After incubation of DendFP and metal ions for 5 min, various concentrations of EDTA or EGTA were added to the solutions of fluorescence-quenched DendFP, and then further incubated for 60 min, at which the fluorescence recovery reached a plateau (Figure 5). With regard to the reversibility of Fe^2+^-induced quenching, the fluorescence emissions were recovered by up to 57.16 and 48.61% when 500 mM EDTA and 100 mM EGTA were added, respectively, whereas less than 3.27% of the fluorescence was recovered at ≤10 mM EDTA and negligible fluorescence recovery (<0.08%) was observed at ≤1 mM EGTA. Regarding the Fe^3+^-induced quenching reversibility, the fluorescence emissions were recovered by up to 69.07 and 76.99% when 500 mM EDTA and 100 mM EGTA were added, respectively, whereas less than 2.09 and 1.36% of the fluorescence was recovered at ≤10 mM EDTA and ≤1 mM EGTA, respectively. Finally, regarding the Cu^2+^-induced quenching reversibility, the fluorescence emissions were recovered by up to 70.77 and 73.30% when 500 mM EDTA and 100 mM EGTA were added, respectively, whereas less than 0.16 and 0.26% of the fluorescence was recovered at ≤10 mM EDTA and EGTA, respectively.

As above, the DendFP fluorescence was restored after the EDTA or EGTA treatments, indicating that these compounds were capable of chelating the metals from the protein. This further implies that metal ions possibly bind to the outer surface of the β-barrel structure of DendFP, rather than to its inner surface. The fluorescence of quenched DendFP by Fe^3+^ and Cu^2+^ was restored by up to approximately 70% by the treatments of both EDTA and EGTA while its final reversibility quenched by Fe^2+^ was significantly lower (Figure 5). This might be because the utilized chelating agents have higher affinities for Fe^2+^ and Fe^3+^. The fluorescence quenched by Fe^2+^ and Fe^3+^ started to be restored by EGTA at low concentrations, such as 5 and 10 mM, where EDTA had little effect. Moreover, when treated with EGTA, maximal recovery could be achieved with its lower concentrations for all the tested metals compared to the case of EDTA. This indicates EGTA was a more effective chelator for the restoration of the metal-quenched DendFP fluorescence.

### 2.4. Selectivity

**Figure 6:**
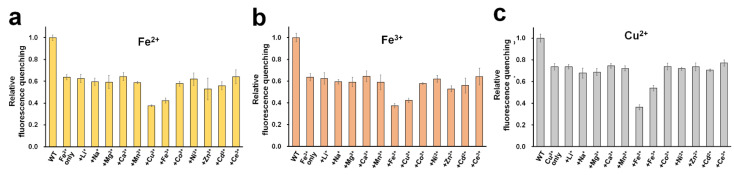
Selectivity. A mixture of (**a**) Fe^2+^, (**b**) Fe^3+^, or (**c**) Cu^2+^ (25 μM) and various metal ions (25 μM) was incubated with DendFP solutions (4 μM). For the control reaction, a solution containing only Fe^2+^, Fe^3+^, or Cu^2+^ without interference metals was used. WT indicates a solution containing only DendFP. Data represent the means ± standard deviations of three replicates.

In addition to Fe^2+^, Fe^3+^, and Cu^2+^, other tested metal ions exhibited fluorescence quenching (Figure 2c). We examined the selectivity to identify whether these metal ions might interfere with the sensing process of DendFP for Fe^2+^, Fe^3+^, and Cu^2+^ (Figure 6). For this, the fluorescence intensity of DendFP was measured after incubating it in the presence of a mixture of each analyte metal (i.e., Fe^2+^, Fe^3+^, or Cu^2+^) and various interference metals. Generally, the addition of the interference metals did not cause a dramatic difference in the fluorescence quenching for DendFP induced by Fe^2+^, Fe^3+^, or Cu^2+^, although Zn^2+^ and Cd^2+^ interfered with Fe^2+^ and Fe^3+^ sensing with small degrees. These results indicate that DendFP is highly selective for Fe^2+^, Fe^3+^, and Cu^2+^. However, there was low selectivity among Fe^2+^, Fe^3+^, and Cu^2+^. Future study is required to identify the specific metal ion for DendFP quenching to utilize it as the more feasible metal biosensor.

### 2.5. Limits of Detection and Quantification

The limits of detection (LOD) and quantification (LOQ) are defined as the lowest concentration of analyte that can be detected and quantified, yielding a signal-to-ratio of 3 and 10, respectively. They are good indicators of a sensor’s performance [33,34]. To determine these values, linear plots were constructed depicting the relationship between the fluorescence quenching (*F*_o_–*F*) and metal ion concentration (Supplementary Figure S1), from which the linear functions for each metal ion (i.e., Fe^2+^, Fe^3+^, and Cu^2+^) were obtained [34]. The standard deviation was calculated from 20 replicates of blank sample (i.e., DendFP solution without metal ions), which was then multiplied by 3 (in the case of LOD) or 10 (in the case of LOQ). Finally, the LOD and LOQ values were determined based on the obtained regression equations in which LODs for Fe^2+^, Fe^3+^, and Cu^2+^ were 3.0, 6.8, and 3.2 μM, respectively, and LOQs for Fe^2+^, Fe^3+^, and Cu^2+^ were 21.5, 32.8, and 14.4 μM, respectively.

### 2.6. Crystal Structure of DendFP

To better understand the molecular mechanism of fluorescence quenching by Fe^2+^, Fe^3+^, and Cu^2+^, we performed co-crystallization of DendFP with metal ions. However, the proteins precipitated when the metal ions were added, and they did not crystallize. Next, DendFP crystals were soaked in a crystal reservoir supplemented with the metal ions, and quenching of the DendFP crystals was clearly observed, indicating the fluorescence-quenched state based on our biochemical study (Figure 2b). However, the DendFP crystals soaked with the metals exhibited no or very poor X-ray diffraction. This possibly occurred because the crystal lattice was damaged, as the metals adhered to the β-barrel surface of DendFP and these metal-bound DendFP molecules interacted with each other. Thus, we focused on determining the native crystal structure of DendFP to investigate its metal-binding sites via comparison with those of previously reported quenchable metal-bound FPs. The DendFP crystal belongs to the orthorhombic space group P2_1_2_1_2_1_, with *a* = 115.826 Å, *b* = 124.737 Å, and *c* = 129.059 Å (Table 1).

**Figure 7:**
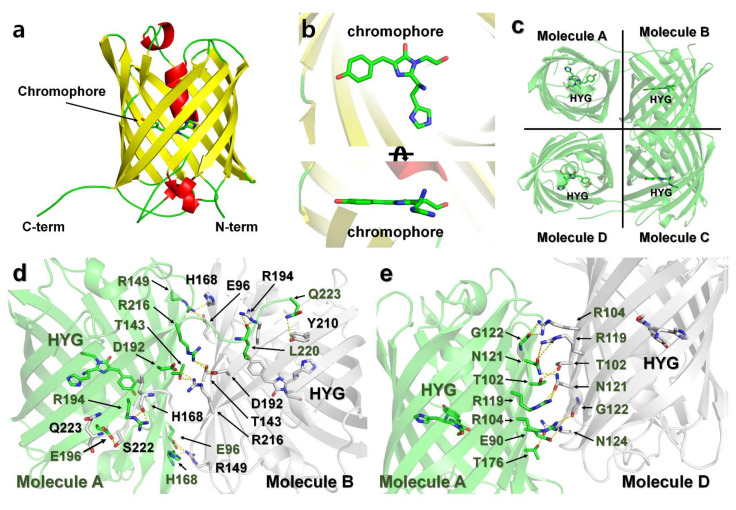
Crystal structure of DendFP. (**a**) Monomer structure of DendFP. (**b**) The chromophore of DendFP comprises the His62-Try63-Gly64 tripeptide (HYG). (**c**) Tetrameric structure of DendFP. Dimeric interface of molecules (**d**) A–B and (**e**) A–D.

The final model of DendFP was refined to a 2.60 Å resolution, with *R*_work_ and *R*_free_ of 23.42 and 28.13%, respectively. DendFP possesses a typical β-barrel fold (Figure 7a), similar to other GFP-like fluorescent proteins. The chromophore of DendFP consisted of the tripeptide His62-Try63-Gly64 located inside the β-barrel and showed a nearly planar conformation with a cis-configuration (Figure 7b). There are eight DendFP molecules in the asymmetric unit, which represent two tetrameric DendFP. The 8 DendFP molecules in the asymmetric unit are almost identical, with an r.m.s. deviation ranging between 0.154 and 0.250 Å. Moreover, the 2 tetrameric DendFP molecules are also almost identical, with an r.m.s. deviation of 0.335 Å. In each tetrameric formation of DendFP, the two dimer molecules are related by a non-crystallographic two-fold pseudosymmetry axis perpendicular to the β-barrel (Figure 7c).

In its tetrameric formation, metal ions cannot access the interface of each molecule, which represents a site where metals cannot bind to DendFP. Accordingly, the tetrameric interfaces of DendFP were analyzed to better understand its putative metal-binding sites. The surface of molecule A (10453 Å^2^) was buried by 13.9% (1459.9 Å^2^) and 30% (806.6 Å^2^) by molecules B and C, respectively (Figure 7c). The DendFP monomer is composed of 219 amino acids. Among these amino acids, 38 residues are located at the A–B interface, which is stabilized by 16 hydrogen bonds and 7 salt bridges (Supplementary Table S1), and 21 residues are located at the A–D interface, which is stabilized by 8 hydrogen bonds (Supplementary Table S1). In particular, the metal ions cannot bind to amino acids (Glu90, Glu96, Thr102, Arg104, Arg119, Asn121, Thr143, Arg149, His168, Thr176, Asp192, Arg194, Arg194, Glu196, Arg216, Ser222, and Gln223) in the A–B and A–D interfaces.

### 2.7. Comparison of Metal-Binding Sites on the Surface of FPs

**Figure 8:**
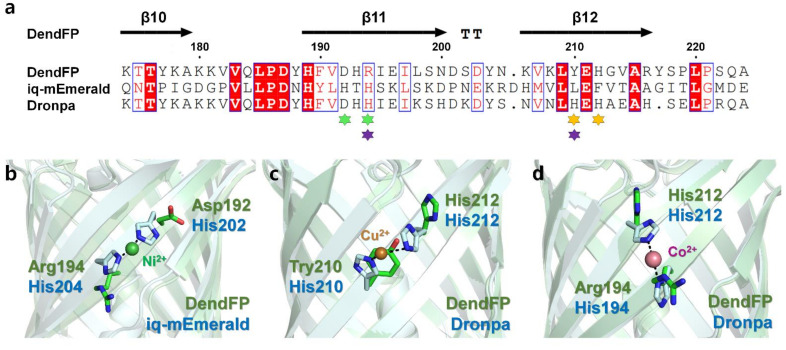
Comparison of the metal-binding sites of DendFP with those of iq-mEmerald and Dronpa. (**a**) Sequence alignments of DendFP with iq-mEmerald and Dronpa. The metal-binding sites of iq-mEmerald (green, Ni^2+^) and Dronpa (orange for Cu^2+^; purple for Co^2+^/Ni^2+^) are indicated by an asterisk. Superimposition of DendFP with (**b**) Ni^2+^-bound iq-mEmerald (PDB code: 4KW4), (**c**) Cu^2+^-bound Dronpa (5HZT), and (**d**) Co^2+^-bound Dronpa (5HZS).

The metal-bound crystal structures of two FPs, iq-mEmerald and Dronpa, have been reported, in which metal ions interacted with two histidine residues on the β-barrel surface of the FPs [17,23]. To better understand the metal-binding site of DendFP, the amino acid sequence and crystal structure of DendFP were comparatively analyzed against iq-mEmerald and Dronpa (Figure 8a). iq-mEmerald was engineered based on the robust transition metal-binding modes (*i* and *i* + 2) [23]. The sequence identity between DendFP and iq-mEmerald is 25.85%, and superimposing both structures showed a similarity with an r.m.s. deviation of 0.998 Å. iq-mEmerald showed the highest fluorescence quenching by Cu^2+^ and exhibited a reduction in fluorescence emission by Co^2+^ and Ni^2+^, whereas Zn^2+^ increased its fluorescence intensity. The Cu^2+^-bound state of iq-mEmerald has not been determined, whereas Ni^2+^- and Zn^2+^-bound states have been reported; these metal ions are bound to engineered His202 and His204 [23]. As a result, it was shown that a robust transition metal-binding site can bind to both fluorescence-inhibiting and -enhancing metal ions. The metal-binding sites His202 and His204 of iq-mEmerald are located at the β11-strand while Asp192 and Arg194 are located at the same structural positions in DendFP; thus, it can be stated that the metal-binding sites are not shared between DendFP and iq-mEmerald (Figure 8a,b).

DendFP has a sequence identity of 70.42% with Dronpa, and the superimposing of both structures showed a similarity with an r.m.s. deviation of 0.374 Å. The metal-binding site of Dronpa is present naturally, without the need for amino acid engineering. Two metal-binding modes were observed in the crystal structure of metal-bound Dronpa [17]. One is His210 and His212 in the β12-strand, the robust metal-binding site mode, which binds most sensitively to Cu^2+^ (Figure 8c). Another metal-binding mode consisted of His194 in the β11-strand and His212 in the β12-strand, to which Co^2+^ or Ni^2+^ were bound (Figure 8d). Following the superimposition of the crystal structures of DendFP and Dronpa, the metal-binding sites His194, His210, and His212 of Dronpa were structurally identical to Arg194, Tyr210, and His212 of DendFP, respectively (Figure 8b,c). Although DendFP did not have a metal-binding site consisting of two identical histidine pairs, such as the case for Dronpa, since His212 of DendFP is sequentially and structurally identical to His212 of Dronpa, our superimposition analysis provides useful information for the optimal engineering of metal-binding sites in DendFP. Meanwhile, there are eight histidine residues in the amino acid sequence of DendFP. We confirmed that there are no robust metal-binding modes (*i* and *i* + 2) in DendFP, unlike the metal-binding site of iq-mEmerald or the Cu^2+^-binding site of Dronpa. Moreover, we investigated the presence of a histidine pair between two β-strands, such as the Co^2+^/Ni^2+^-binding mode in Dronpa, but there no such identical binding mode was observed in DendFP. Thus, the metal-binding sites of DendFP is different from the previously reported metal-binding sites of iq-mEmerald or Dronpa.

## 3. Discussion

Here, we performed a spectroscopic and structural analysis of the fluorescence quenching of DendFP by metal ions. Previous spectroscopic analysis has shown that most fluorescent proteins, such as DsRed, iq-mEmerald, Dronpa, AmCyan, mOrange, and ZsYellow, are highly sensitive to Cu^2+^ [17,18,19], whereas DendFP is more sensitive to Fe^2^^+^ and Fe^3+^ than Cu^2+^, which is similar to ZsGreen [25]. The dissociation constant of DendFP for Fe^2+^ (24.59 μM), Fe^3+^ (41.66 μM), and Cu^2+^ (137.18 μM) showed a similar order of sensitivity to ZsGreen for Fe^2+^ (11.5 μM), Fe^3+^ (16.3 μM), and Cu^2+^ (68.3 μM). However, DendFP showed a maximum binding capacity of >100 in the cases of Fe^2+^, Fe^3+^, and Cu^2+^; however, for ZsGreen, the maximum binding capacity of Fe^2+^ and Fe^3+^ was >100, whereas that of Cu^2+^ was 82.9. Although the overall quenching performances of DendFP for Fe^2+^ and Fe^3+^ were superior to that for Cu^2+^ when evaluated based on the Langmuir isotherm, the DendFP fluorescence was quenched by Cu^2+^ more than by Fe^2+^ or Fe^3+^ at the low concentrations, such as 5 μM. Such a discrepancy could be because Cu^2+^ binding does not strictly follow the Langmuir model based on monolayer binding.

Fluorescence quenching typically involves two distinct mechanisms: collisional quenching and static quenching [33,35,36]. A simple approach to distinguish the two systems is to observe the alteration in the *K*_sv_ values at different temperatures. In theory, in collisional quenching, a higher temperature increases the frequency of collision between the fluorescence chromophore in its excited state and a quencher, resulting in a more sensitive quenching (i.e., greater slope; higher *K*_sv_ value) in the Stern–Volmer plot. This phenomenon is in contrast to that of a static system in which a non-fluorescent complex is generated between the fluorophore and a quencher in the ground state. In static quenching, a less steep slope (i.e., lower *K*_sv_ value) tends to be observed at a higher temperature, which possibly occurs by disrupting the fluorophore–quencher bond interaction. In this study, higher *K*_sv_ values were observed with Fe^2+^ and Cu^2+^ by increasing the temperature although the difference was not prominent, indicating that Fe^2+^- and Cu^2+^-induced fluorescence quenching of DendFP might occur based on the collisional mechanism. Absorption spectra could also provide useful insight into the quenching process (Supplementary Figure S2) [36]. In Cu^2+^-induced quenching, its presence at different concentrations did not affect the spectra, suggesting it is a collisional process. In contrast, both Fe^2+^ and Fe^3+^ caused a spectral change at high concentrations (i.e., 500 and 5000 μM). Such a spectral change may be an indication of static processes for Fe ions, which is inconsistent with the results from the Stern–Volmer plot (Figure 4). For definitive elucidation, a fluorescence lifetime measurement study of DendFP is needed in the future.

The reversibility of DendFP for Fe^2+^ (22.17%), Fe^3+^ (11.93%), and Cu^2+^ (45.15%) was distinguished from that of ZsGreen for Fe^2+^ (15.86%), Fe^3+^ (13.29%), and Cu^2+^ (89.47%), upon addition of 50 mM EDTA. Therefore, fluorescence quenching of DendFP by metal ions shows unique optical properties when compared with previously reported FPs. Although the fluorescence of metal-quenched DendFP was not fully recovered, its reversibility observed in this study still provides the possibility of the utilization of the protein as a reusable metal biosensor and insights into the location of metal sites in the protein.

The identification of a quenchable metal-binding site of DendFP can not only explain the mechanism of action in fluorescence quenching but can also provide important information to engineer FPs with high sensitivity to specific metals in the application stage. In this study, DendFP fluorescence was quenched by Fe and Cu ions, and when DendFP was directly applied as a metal biosensor, its fluorescence quenching was induced by both Fe and Cu ions. In this case, it is difficult to determine which of the two ions actually quenched DendFP. Thus, if specific metal-binding sites on DendFP are found, it is possible to mutate the binding sites of other metal ions to detect only the desired metal. In this study, the crystal structure of the tetrameric interface of DendFP was analyzed. Our findings provide information on the sites that are potentially unable to bind to the metal ions. The metal-binding sites of the DendFP crystal structure were compared to those of iq-mEmerald, and Dronpa. DendFP is considered to be involved in metal binding via other types of amino acids, unlike the previously described metal-binding modes of FPs, which utilize two histidines. To identify the metal-binding sites of DendFP, it will be necessary to construct an improved crystallization method to investigate the quenchable metal-bound state of DendFP. Since FP-based metal biosensors are intended to detect specific metals in unknown samples, the procedure will require overall screening for specific metals and an examination of the extended effects of metals on the fluorescence quenching of DendFP. Here, we analyzed the fluorescence quenching properties by metals specific for purified DendFP. Moreover, since this protein can be expressed by cells, it could be used to monitor the metal fluctuations in a cellular environment, as previously reported in an in vivo metal quenching experiment using a VAMP2/iq-mApple/mEmerald probe [23].

In summary, our results not only elucidate the properties of the metal-based fluorescence quenching of DendFP but also provide insights for the development of FP-based metal biosensors in the future.

## 4. Materials and Methods

### 4.1. Protein Preparation

The codon-optimized DendFP (Uniprot: Q8T6U0) was synthesized and cloned into the pET-28a vector. The recombinant DNA was transformed into *Escherichia coli* BL21 (DE3). Cells were incubated with vigorous shaking at 200 rpm at 37 °C. When OD_600_ reached 0.6–0.8, protein expression was induced by adding 0.5 mM isopropyl β-d-1-thiogalactopyranoside (IPTG), and the culture was incubated overnight at 20 °C. After cell harvesting by centrifugation, the pellet was resuspended in lysis buffer (50 mM Tris-HCl, pH 8.0, 200 mM NaCl, and 20 mM imidazole). After cell lysis on ice by sonication, the cell debris was removed by centrifugation at 14,000 rpm for 30 min. The supernatant was loaded onto an Ni-NTA column (Qiagen, Hilden, Germany). After washing the resin using lysis buffer, the protein was eluted using a buffer containing 50 mM Tris-HCl, pH 8.0, 200 mM NaCl, and 300 mM imidazole. To remove the N-terminal hexahistidine-tagged recombinant protein, thrombin was added into the eluted faction, followed by incubation at room temperature overnight. The cleavage of the expression-tag in recombinant DendFP was verified by 15% SDS-PAGE. This protein was concentrated using an Amicon concentrator (Merck, Kenilworth, NJ, USA, cutoff: 10 kDa) and loaded on a Sephacryl 100-HR column (GE Healthcare, Chicago, IL, USA) equilibrated with 10 mM Tris-HCl pH 8.0 and 200 mM NaCl. Protein concentrations were measured by the Bradford assay using a Synergy H1 microplate reader (BioTek, Winooski, VT, USA) at 25 °C.

### 4.2. Spectroscopic Analysis

Purified DendFP were verified by the spectra scan method through measurement of the maximum fluorescence excitation and emission wavelengths in the ranges of 400–600 and 450–640 nm, respectively. The fluorescence emission spectra of DendFP in the presence of difference concentrations of Fe^2+^, Fe^3+^, and Cu^2+^ at 0, 5, 50, 500, and 5000 μM were also measured in the ranges of 450–640 nm. In the fluorescence emission measurement experiment, the protein and metal mixture were excited at a wavelength of 480 nm, and the fluorescence emission was measured at a wavelength of 530 nm. All samples were placed in a 96-well plate for fluorescence measurement, and before measurement, all mixed samples were shaken in the orbital direction for 10 s. Fluorescence emission was measured using a Synergy H1 microplate reader (BioTek). All experiments were performed in triplicate at 25 °C. The fluorescence intensity values were not corrected due to negligible inner-filter effects.

### 4.3. Screening Metal-Induced Quenching

Metal ion screening was performed using a 10 μM solution of purified DendFP and 10 mM solutions of LiCl, NaCl, MgCl_2_, CaCl_2_, MnCl_2_, FeCl_2_, FeCl_3_, CoCl_2_, NiCl_2_, CuCl_2_, ZnCl_2_, CdCl_2_, and CeCl_3_. For the visualization of fluorescence quenching, 50 μL of DendFP solution and 50 μL of each metal solution were mixed in a PCR tube and incubated at room temperature for 5 min. Each mixture sample was place on an LED transilluminator and exposed to LED light with a wavelength of 470 nm. To quantify the fluorescence intensity, the same DendFP and metal ion mixtures were prepared; the fluorescence emission of the mixtures was measured 5 min after the reaction.

### 4.4. Quenchable Metal Titration

Fe^2+^, Fe^3+^, and Cu^2+^ titration experiments were carried out to determine the dissociation constant (*K*_d_) and maximum binding capacity (*B*_max_) of the metal ions for DendFP. The purified 4 μM DendFP solution (50 μL) was mixed with an equal volume of various concentrations of FeCl_2_, FeCl_3_, or CuCl_2_ solutions ranging from 0 to 10,000 μM, followed by incubation at 25 °C for 5 min. Then, the fluorescence emission of the mixtures was measured at a wavelength of 530 nm.

The relative fluorescence quenching (%) was calculated based on the fluorescence emission of native DendFP. The titration results for Fe^2+^, Fe^3+^, and Cu^2+^ were analyzed using the SigmaPlot 12.3 software (Systat Software, Erkrath, Germany).

The Langmuir equation was used for data fitting:

$$\mathrm{Relative}\;\mathrm{fluorescence}\;\mathrm{quenching}\;\left(\%\right)=\frac{B_{\max}\;{\left[M\right]}_{\mathrm{unbound}}}{K_{d}+{\left[M\right]}_{\mathrm{unbound}}}$$

where *K_d_* indicates the equilibrium dissociation constant (μM) and *B*_max_ indicates the maximum binding capacity or the concentration of available binding sites of a molecule. [*M*]_unbound_ is the equilibrium concentration of unbound metal ions (μM). When [*M*]_total_ is present in a large molar excess relative to that of DendFP, [*M*]_unbound_ is assumed to be equal to [*M*]_total_. As the amount of metal ions bound to DendFP was represented by relative fluorescence quenching (%) in this study, *B*_max_ was accordingly defined as the maximum registered fluorescence [32].

### 4.5. Reversibility of Metal-Induced Fluorescence Quenching

To investigate the reversibility of DendFP metal-induced fluorescence quenching, we prepared the fluorescence-quenched state of DendFP by mixing 50 μL of DendFP (4 μM) with 50 μL of each metal solution (1 mM Fe^2+^, 1 mM Fe^3+^, or 10 mM Cu^2+^), followed by incubation for 5 min at 25 °C. The quenched state of each mixture was verified by measuring its fluorescence emission. Then, 50 μL of EDTA (1, 5, 10, 50, 100, and 500 mM) or EGTA (1, 5, 10, 50, 100, and 200 mM) solution were added to the quenched solution. After incubation for 60 min at 25 °C, the fluorescence emission was measured at a wavelength of 530 nm.

### 4.6. Selectivity of DendFP Sensing for Fe^2+^, Fe^3+^, and Cu^2+^

To examine the selectivity of DendFP for Fe^2+^, Fe^3+^, and Cu^2+^, 4 μM of DendFP (50 μL) was mixed with a solution containing 25 μM of Fe^2+^, Fe^3+^, or Cu^2+^ (25 μL) and 25 μM of various interference metal ions (25 μL), such as LiCl, NaCl, MgCl_2_, CaCl_2_, MnCl_2_, CoCl_2_, NiCl_2_, ZnCl_2_, CdCl_2_, and CeCl_3_. For the control reaction, a solution containing only Fe^2+^, Fe^3+^, or Cu^2+^ without interference metals was used. The reaction mixture was incubated for 5 min at 25 °C, which was then subjected to fluorescence measurement.

### 4.7. Crystallization and X-ray Data Collection

Purified DendFP was concentrated to 20 mg/mL for crystallization using an Amicon concentrator (Merck, cutoff: 10 kDa). Protein crystallization was performed in 24-well plates using the hanging drop vapor diffusion method at 22 °C. Drops consisting of 1 µL of protein solution and 1 µL of reservoir solution consisting of 100 mM Tris-HCl, pH 7.5, 0.2 M MgCl_2_, and 20% PEG4000 were mixed and equilibrated over 300 µL. Suitable crystals for X-ray diffraction were grown within 5–7 days. X-ray diffraction data were collected from the beamline 11C at the Pohang Accelerator Laboratory (Pohang, Korea) [37]. The DendFP crystals were immersed in cryoprotectant solution, which consisted of the reservoir solution supplemented with 20% (*w*/*v*) glycerol, and immediately placed under a liquid nitrogen stream at 100 K. Diffraction data were recorded with the Pilatus 6M detector and processed using the HKL2000 program [38]. The data statistics are shown in Table 1.

### 4.8. Structure Determination and Analysis

The electron density map was obtained by the molecular replacement method with the MOLREP [39] program using the crystal structure of DendFP (PDB code: 5EXB) as a search model [26]. The model building and refinement were performed with Coot [40] and REFMAC5 [41], respectively. The geometry of the final model was checked using MolProbity [42]. The structure figure was visualized with PyMOL (http://pymol.org/, accessed on 3 May 2022). The tetrameric interfaces of DendFP were analyzed with PISA [43]. Protein sequence alignments were performed using ClustalW [44] and visualized with ESPript [45].

## Figures and Tables

**Table 1:** Data collection and refinement statistics for DendFP.

Data Collection	DendFP
Space group	P2_1_2_1_2_1_
Cell dimensions	
*a*, *b*, *c* (Å)	115.826, 124.737, 129.059
Resolution (Å)	50.0–2.6 (2.64–2.60)
Completeness	95.5 (89.3)
Redundancy	4.2 (2.7)
I/σ(I)	10.6 (1.6)
*R*_merge_ (%) ^a^	0.136 (0.357)
Refinement statistics	
Resolution (Å)	48.70–2.60
*R*_work_ (%) ^b^	23.42
*R*_free_ (%) ^c^	28.13
B-factor (Averaged)	
Protein	42.88
R.m.s deviations	
Bond lengths (Å)	0.006
Bond angles (°)	1.634
Ramachandran plot (%)	
favored	97.9
Allowed	2.1

Highest resolution shell is shown in parentheses. ^a^ *R*_merge_ = Σ*_h_*Σ*_i_*|I*i*(hkl)_<*I*(hkl)>|/Σ*_h_*Σ*_i_*I*_i_*(hkl), where *I_i_*(hkl) is the intensity of the ‘ith’ measurement of reflection hkl and <*I*(hkl)> is the weighted mean of all measurements of hkl. ^b^ *R*_work_ = Σ||*F*_obs_|−|*F*_calc_||/Σ|*F*_obs_|, where *F*_obs_ and *F*_calc_ are the observed and calculated structure–factor amplitudes respectively. ^c^ *R*_free_ was calculated as *R*_work_ using a randomly selected subset of unique reflections not used for structure refinement.

## Data Availability

The atomic coordinates and the structure factors for DendFP (PDB ID: 7DIG) have been deposited in the Protein Data Bank.

## Supplementary Materials

The following supporting information can be downloaded at: https://www.mdpi.com/article/10.3390/molecules27092922/s1, Figure S1: The linear plot between fluorescence quenching of DendFP and concentration of metal ions; Figure S2: Absorbance spectra of DendFP solution in the absence and presence of metal ions; Table S1: Interactions on the dimeric A-B interface of DendFP; Table S2: Interactions on the dimeric A-D interface of DendFP.

## Sample Availability

Samples of the compounds are not available from the authors.

## References

[1] Verma N., Singh M. (2005). Biosensors for heavy metals. Biometals.

[2] Rigo A.A., Cezaro A.M., Muenchen D.K., Martinazzo J., Manzoli A., Steffens J., Steffens C. (2020). Heavy metals detection in river water with cantilever nanobiosensor. J. Environ. Sci. Health B.

[3] Tan S.S., Kim S.J., Kool E.T. (2011). Differentiating between fluorescence-quenching metal ions with polyfluorophore sensors built on a DNA backbone. J. Am. Chem. Soc..

[4] Mehta J., Bhardwaj S.K., Bhardwaj N., Paul A.K., Kumar P., Kim K.H., Deep A. (2016). Progress in the biosensing techniques for trace-level heavy metals. Biotechnol. Adv..

[5] Tsien R.Y. (1998). The green fluorescent protein. Annu. Rev. Biochem..

[6] Remington S.J. (2011). Green fluorescent protein: A perspective. Protein Sci..

[7] Zimmer M. (2002). Green fluorescent protein (GFP): Applications, structure, and related photophysical behavior. Chem. Rev..

[8] Nam K.H., Kwon O.Y., Sugiyama K., Lee W.H., Kim Y.K., Song H.K., Kim E.E., Park S.Y., Jeon H., Hwang K.Y. (2007). Structural characterization of the photoswitchable fluorescent protein Dronpa-C62S. Biochem. Biophys. Res. Commun..

[9] Xu Y., Hwang K.Y., Nam K.H. (2018). Spectral and structural analysis of large Stokes shift fluorescent protein dKeima570. J. Microbiol..

[10] Kim S.E., Hwang K.Y., Nam K.H. (2019). Spectral and structural analysis of a red fluorescent protein from Acropora digitifera. Protein Sci..

[11] Seward H.E., Bagshaw C.R. (2009). The photochemistry of fluorescent proteins: Implications for their biological applications. Chem. Soc. Rev..

[12] Saeed S., Mehreen H., Gerlevik U., Tariq A., Manzoor S., Noreen Z., Sezerman U., Bokhari H. (2020). HriGFP novel flourescent protein: Expression and applications. Mol. Biotechnol..

[13] Bae J.E., Kim I.J., Nam K.H. (2017). Disruption of the hydrogen bonding network determines the pH-induced non-fluorescent state of the fluorescent protein ZsYellow by protonation of Glu221. Biochem. Biophys. Res. Commun..

[14] Lee W., Kim H., Kang Y., Lee Y., Yoon Y. (2019). A biosensor platform for metal detection based on enhanced green fluorescent protein. Sensors.

[15] Martinez A.R., Heil J.R., Charles T.C. (2019). An engineered GFP fluorescent bacterial biosensor for detecting and quantifying silver and copper ions. Biometals.

[16] Eli P., Chakrabartty A. (2006). Variants of DsRed fluorescent protein: Development of a copper sensor. Protein Sci..

[17] Kim I.J., Kim S., Park J., Eom I., Kim S., Kim J.H., Ha S.C., Kim Y.G., Hwang K.Y., Nam K.H. (2016). Crystal structures of Dronpa complexed with quenchable metal ions provide insight into metal biosensor development. FEBS Lett..

[18] Bae J.E., Kim I.J., Nam K.H. (2018). Spectroscopic analysis of the Cu(2+)-induced fluorescence quenching of fluorescent proteins AmCyan and mOrange2. Mol. Biotechnol..

[19] Kim I.J., Xu Y., Nam K.H. (2020). Spectroscopic and structural analysis of Cu(2+)-induced fluorescence quenching of ZsYellow. Biosensors.

[20] Jiang S.D., Sheng Y., Wu X.J., Zhu Y.L., Li P.P. (2021). Chromophorylation of a novel cyanobacteriochrome GAF domain from spirulina and its response to copper ions. J. Microbiol. Biotechnol..

[21] Sumner J.P., Westerberg N.M., Stoddard A.K., Hurst T.K., Cramer M., Thompson R.B., Fierke C.A., Kopelman R. (2006). DsRed as a highly sensitive, selective, and reversible fluorescence-based biosensor for both Cu(+) and Cu(2+) ions. Biosens. Bioelectron..

[22] Peterffy J.P., Szabo M., Szilagyi L., Lanyi S., Abraham B. (2015). Fluorescence of a histidine-modified enhanced green fluorescent protein (EGFP) effectively quenched by copper(II) ions. Part II. Molecular determinants. J. Fluoresc..

[23] Yu X., Strub M.P., Barnard T.J., Noinaj N., Piszczek G., Buchanan S.K., Taraska J.W. (2014). An engineered palette of metal ion quenchable fluorescent proteins. PLoS ONE.

[24] Barondeau D.P., Kassmann C.J., Tainer J.A., Getzoff E.D. (2002). Structural chemistry of a green fluorescent protein Zn biosensor. J. Am. Chem. Soc..

[25] Kim I.J., Xu Y., Nam K.H. (2021). Spectroscopic analysis of Fe ion-induced fluorescence quenching of the green fluorescent protein ZsGreen. J. Fluoresc..

[26] Pletneva N.V., Pletnev S., Pakhomov A.A., Chertkova R.V., Martynov V.I., Muslinkina L., Dauter Z., Pletnev V.Z. (2016). Crystal structure of the fluorescent protein from *Dendronephthya* sp. in both green and photoconverted red forms. Acta Crystallogr. D Struct. Biol..

[27] Pakhomov A.A., Chertkova R.V., Martynov V.I. (2015). Ph-sensor properties of a fluorescent protein from *Dendronephthya* sp.. Bioorg. Khim..

[28] Magrane J., Cortez C., Gan W.B., Manfredi G. (2014). Abnormal mitochondrial transport and morphology are common pathological denominators in SOD1 and TDP43 ALS mouse models. Hum. Mol. Genet..

[29] McCamphill P.K., Ferguson L., Sossin W.S. (2017). A decrease in eukaryotic elongation factor 2 phosphorylation is required for local translation of sensorin and long-term facilitation in Aplysia. J. Neurochem..

[30] Pedersen M., Jamali S., Saha I., Daum R., Bendjennat M., Saffarian S. (2019). Correlative iPALM and SEM resolves virus cavity and Gag lattice defects in HIV virions. Eur. Biophys. J..

[31] Bougie I., Charpentier S., Bisaillon M. (2003). Characterization of the metal ion binding properties of the hepatitis C virus RNA polymerase. J. Biol. Chem..

[32] Pinto P.C.A.G., Ribeiro D.M.G.P., Azevedo A.M.O., Justina V.D., Cunha E., Bica K., Vasiloiu M., Reis S., Saraiva M.L.M.F.S. (2013). Active pharmaceutical ingredients based on salicylate ionic liquids: Insights into the evaluation of pharmaceutical profiles. New J. Chem..

[33] Wahba M.E.K., El-Enany N., Belal F. (2015). Application of the Stern–Volmer equation for studying the spectrofluorimetric quenching reaction of eosin with clindamycin hydrochloride in its pure form and pharmaceutical preparations. Anal. Methods.

[34] Huang A., Li W., Shi S., Yao T. (2017). Quantitative fluorescence quenching on antibody-conjugated graphene oxide as a platform for protein sensing. Sci. Rep..

[35] Zhao H., Zastrow M.L. (2022). Transition metals induce quenching of monomeric near-infrared fluorescent proteins. Biochemistry.

[36] Isarankura-Na-Ayudhya C., Tantimongcolwat T., Galla H.-J., Prachayasittikul V. (2009). Fluorescent protein-based optical biosensor for copper ion quantitation. Biol. Trace Elem. Res..

[37] Park S.Y., Ha S.C., Kim Y.G. (2017). The protein crystallography beamlines at the pohang light source II. Biodesign.

[38] Otwinowski Z., Minor W. (1997). Processing of X-ray diffraction data collected in oscillation mode. Methods Enzymol..

[39] Vagin A., Teplyakov A. (2010). Molecular replacement with MOLREP. Acta Crystallogr. D Biol. Crystallogr..

[40] Emsley P., Cowtan K. (2004). Coot: Model-building tools for molecular graphics. Acta Crystallogr. D Biol. Crystallogr..

[41] Murshudov G.N., Skubak P., Lebedev A.A., Pannu N.S., Steiner R.A., Nicholls R.A., Winn M.D., Long F., Vagin A.A. (2011). REFMAC5 for the refinement of macromolecular crystal structures. Acta Crystallogr. D Biol. Crystallogr..

[42] Williams C.J., Headd J.J., Moriarty N.W., Prisant M.G., Videau L.L., Deis L.N., Verma V., Keedy D.A., Hintze B.J., Chen V.B. (2018). MolProbity: More and better reference data for improved all-atom structure validation. Protein Sci..

[43] Krissinel E., Henrick K. (2007). Inference of macromolecular assemblies from crystalline state. J. Mol. Biol..

[44] Larkin M.A., Blackshields G., Brown N.P., Chenna R., McGettigan P.A., McWilliam H., Valentin F., Wallace I.M., Wilm A., Lopez R. (2007). Clustal W and Clustal X version 2.0. Bioinformatics.

[45] Gouet P., Courcelle E., Stuart D.I., Metoz F. (1999). ESPript: Analysis of multiple sequence alignments in PostScript. Bioinformatics.

