# Supplementary material for: Metal-Induced Fluorescence Quenching of Photoconvertible Fluorescent Protein DendFP
Source: Molecules. 2022 May 3;27(9):2922. doi: 10.3390/molecules27092922 (PMC9104182; doi:10.3390/molecules27092922)
Supplement: Supplementary file 1 [file molecules-27-02922-s001.zip › molecules-1549399-supplementary.pdf]

Supplementary Information

Metal-induced fluorescence quenching of photoconvertible fluorescent protein DendFP

In Jung Kim [1,†], Yongbin Xu [2,3] and Ki Hyun Nam [4,5,*]

[1] Division of Biotechnology, College of Life Sciences and Biotechnology, Korea University, Seoul 02841, Korea; ij0308@korea.ac.kr

[2] Department of Bioengineering, College of Life Science, Dalian Minzu University, Dalian 116600, China; yongbinxu@dlnu.edu.cn

[3] Key Laboratory of Biotechnology and Bioresources Utilization of Ministry of Education, Dalian Minzu University, Dalian 116024, China; yongbinxu@dlnu.edu.cn

[4] Department of Life Science, Pohang University of Science and Technology, Pohang 37673, Korea

[5] POSTECH Biotech Center, Pohang University of Science and Technology, Pohang 37673, Korea

[†]Current address: Research Institute of Tailored Food Technology, Kyungpook National University, Daegu 41566, Korea

*Correspondence: structures@postech.ac.kr

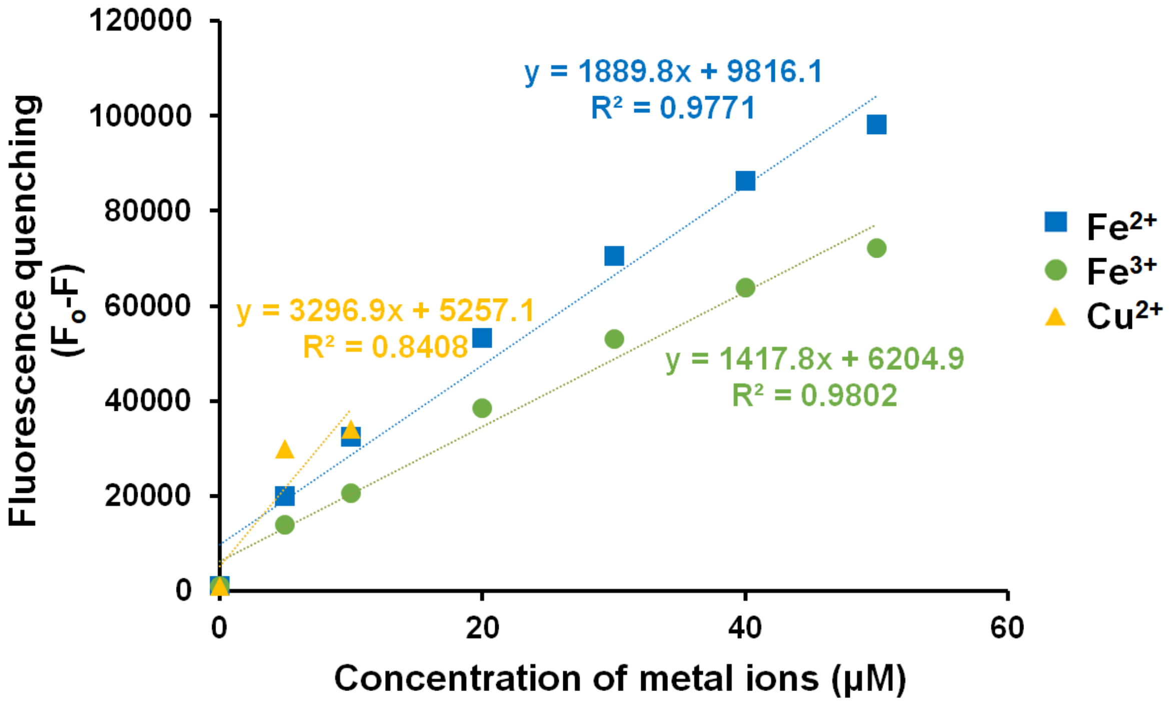

Figure S1. The linear plot between fluorescence quenching of DendFP (F_o-F) and concentration of metal ions (i.e. Fe^{2+}, Fe^{3+}, and Cu^{2+}), generated for LOD and LOQ determination. Data points represent the means of three replicates.

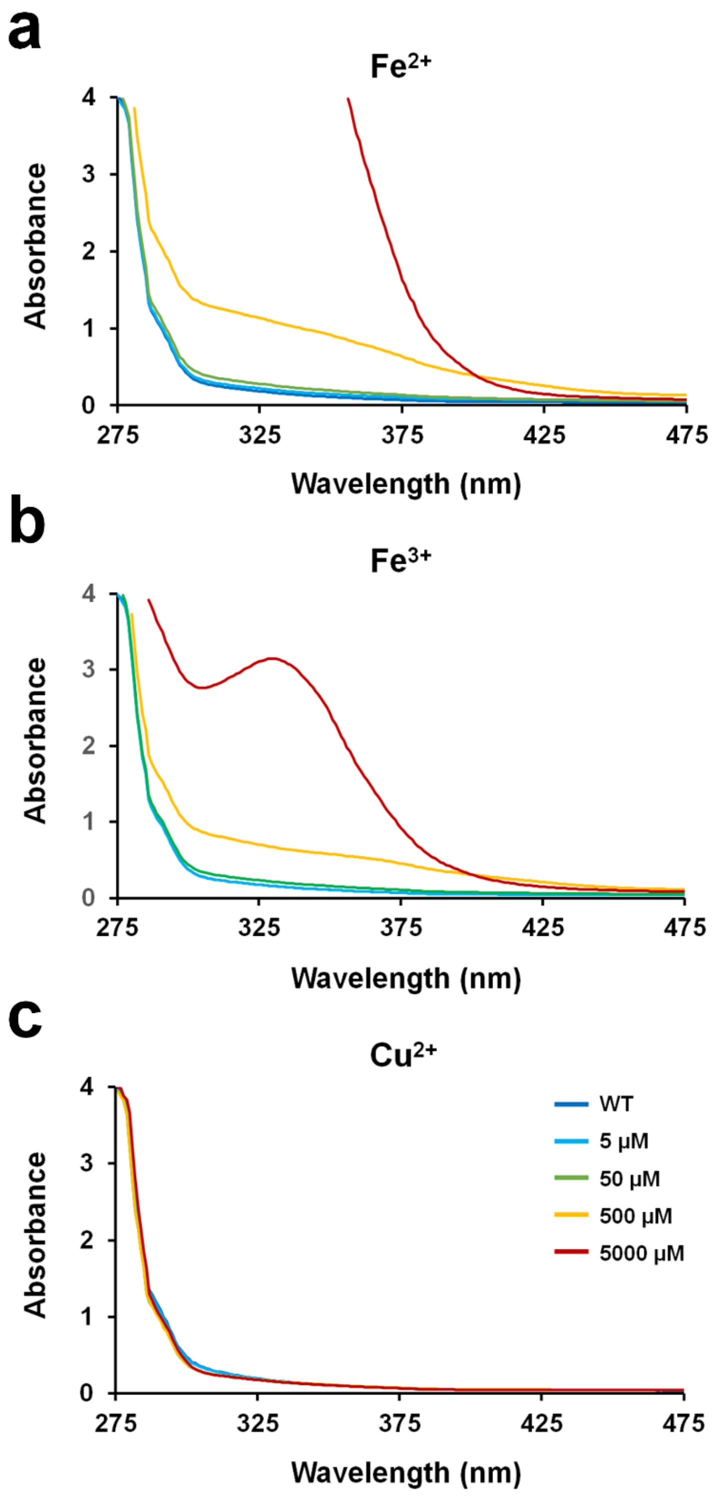

Figure S2. Absorbance spectra of DendFP solution in the absence and presence of (a) Fe^{2+}, (b) Fe^{3+}, and (c) Cu^{2+} at different concentrations (0, 5, 50, 500, and 5000 μM).

Table S1. Interactions on the dimeric A-B interface of DendFP

Molecule A (Residue [atom])	Distance [Å]	Molecule B (Residue [atom])
**Hydrogen bonds**		
GLU96 [OE1]	2.85	ARG149 [NH1]
GLU96 [OE2]	3.48	ARG149 [NH2]
THR143 [OG1]	3.35	ARG216 [NH1]
ARG149 [NH2]	2.66	GLU96 [OE2]
ARG149 [NH2]	3.34	HIS168 [O]
HIS168 [O]	2.70	ARG149 [NH2]
ASP192 [OD1]	3.80	ARG216 [NH1]
ARG194 [NE]	2.73	LEU220 [O]
ARG194 [NH2]	2.80	LEU220 [O]
GLU196 [OE1]	3.06	GLN 223 [N]
GLU196 [OE2]	2.88	SER 222 [OG]
ARG216 [NH1]	3.56	THR143 [OG1]
LEU220 [O]	2.68	ARG194 [NE]
SER222 [OG]	2.38	GLU196 [OE2]
GLN223 [N]	2.89	GLU196 [OE1]
GLN223 [NE2]	3.75	TYR210 [OH]
**Salt Bridges**		
GLU96 [OE1]	2.85	ARG149 [NH1]
GLU96 [OE1]	3.64	ARG149 [NH2]
GLU96 [OE2]	3.48	ARG149 [NH2]
ASP192 [OD1]	3.80	ARG216 [NH1]
ARG149 [NH1]	3.56	GLU96 [OE1]
ARG149 [NH2]	3.25	GLU96 [OE1]
ARG149 [NH2]	2.66	GLU96 [OE2]

Table S2. Interactions on the dimeric A-D interface of DendFP

Molecule A (Residue [atom])	Distance [Å]	Molecule D (Residue [atom])
**Hydrogen bonds**		
GLU90 [OE1]	3.03	ASN124 [N]
THR102 [OG1]	3.76	THR102 [OG1]
ARG104 [NH1]	2.61	GLY122 [O]
ARG119 [NH1]	3.30	ASN121 [OD1]
ARG119 [NH2]	3.54	ASN121 [OD1]
ASN121 [OD1]	3.36	ARG119 [NH1]
GLY122 [O]	3.22	ARG104 [NH1]
THR176 [OG1]	2.87	ASN124 [OD1]
